# A historical overview of *Batrachochytrium dendrobatidis* infection from specimens at the National Zoological Collection Suriname

**DOI:** 10.1371/journal.pone.0239220

**Published:** 2020-10-02

**Authors:** Rawien Jairam

**Affiliations:** Anton de Kom University of Suriname, National Zoological Collection Suriname, Paramaribo, Suriname; Universitat Trier, GERMANY

## Abstract

The amphibian skin disease chytridiomycosis, caused by the pathogenetic fungus *Batrachochytrium dendrobatidis* (Bd) has become one of the major contributors to global amphibian population declines and extinctions. This fungus has spread globally and has caused mortalities in nearly every continent. In South America, Suriname, Guyana and Paraguay are among the remaining three countries where Bd has not been detected to date. To complete the assessment of the possible presence of Bd in Suriname, 205 specimens from the Zoological Collection of Suriname, compromising 6 frog families and 15 genera were sampled for chytrid fungus. No specimens were found to be infected by this fungus and as such the outcome strengthens the previous result of field sampling that there is no support that Bd has spread to Suriname.

## Introduction

Amphibians are generally regarded as good indicators for habitat or ecosystem health due to their dependency on both terrestrial and aquatic habitats for their life history [1]. With 40% of global amphibian species currently at risk of declines or extinctions [https://www.iucn-amphibians.org/; accessed 8 June 2020], concerns for amphibian threats are increasing, with human activities at the forefront of risks [2]. The decline in amphibian populations is not something occurring locally but was demonstrated by [3, 4] to be on a more global scale than once thought. In addition to rapid habitat change from anthropogenic factors, a top contributor to amphibian losses includes the human-mediated introduction of invasive alien species throughout the world including emerging infectious diseases such as chytridiomycosis caused by *Batrachochytrium dendrobatidis* (Bd) [5]. Originating in South Korea [6] this disease-causing skin fungal pathogen has taken proportions of a global epidemic with disease affecting species on all continents with amphibians. In South America, Bd has been detected in amphibian communities within all the countries apart from Suriname, Guyana and Paraguay (Bd-maps.net, accessed December 2019). In Suriname, tests for Bd presence were initially conducted by sampling *Atelopus hoogmoedi* at the Brownsberg Nature Park [7]. In a second study, *Pipa pipa* frogs collected from Suriname and deposited in museums abroad were also been tested for Bd by Soto-Azat et al. [8]; this research was done to verify the possibility of African *Xenopus* spp. as a source of Bd, and also tested other frog species in the family Pipidae from Guyana, Venezuela, French Guiana, and Africa [8]. No Pipidae specimens of these South American countries collected between 1844 and 1994 tested positive for Bd [8]. Although Bd has not been detected in Suriname to date, it has been detected in many places in French Guiana [9] with one location being approximately 7 km away from the border of Suriname where frog infection rates ranged between 1–5% [9]. Though Bd has just recently gathered a lot of attention by the international community, the existence of this fungus can be found in museum specimens dating back as far as the 1970’s [10]. Knowledge of verified gaps in Bd occurrence is of paramount importance, as enhanced biosecurity may forestall inadvertent human-mediated transmission, aiding amphibian conservation. Although initial investigations of selected taxa including one field study [7] and one museum study [8] have not supported Bd presence in Suriname, additional sampling is warranted for broader spatial and taxonomic coverage. Herein, we report findings of a broader geographic and taxonomic study in Suriname, conducted by checking museum specimens housed at the National Zoological Collection Suriname (NZCS) for the presence of Bd. The advantages of testing museum specimens for the presence of Bd includes a provision of an historical overview of the time that the fungus is present in a particular species and in a particular location. Despite the fact that most museum specimens are formalin fixed, successful studies have still been able to check for the presence of Bd [11–14]. The few museum specimens from Suriname tested by Soto-Azat et al. [8] in a single family support the efficacy to test more species using this approach. Additionally, it allowed us to examine the Bd presence across widespread locations that could be logistically constrained today due to budgetary and time limitations for sampling species in the wild. The research presented herein gives an overview of specimens tested for Bd stored at the NZCS in 6 families and 15 genera compromising 205 specimens.

## Materials and methods

**Fig 1:**
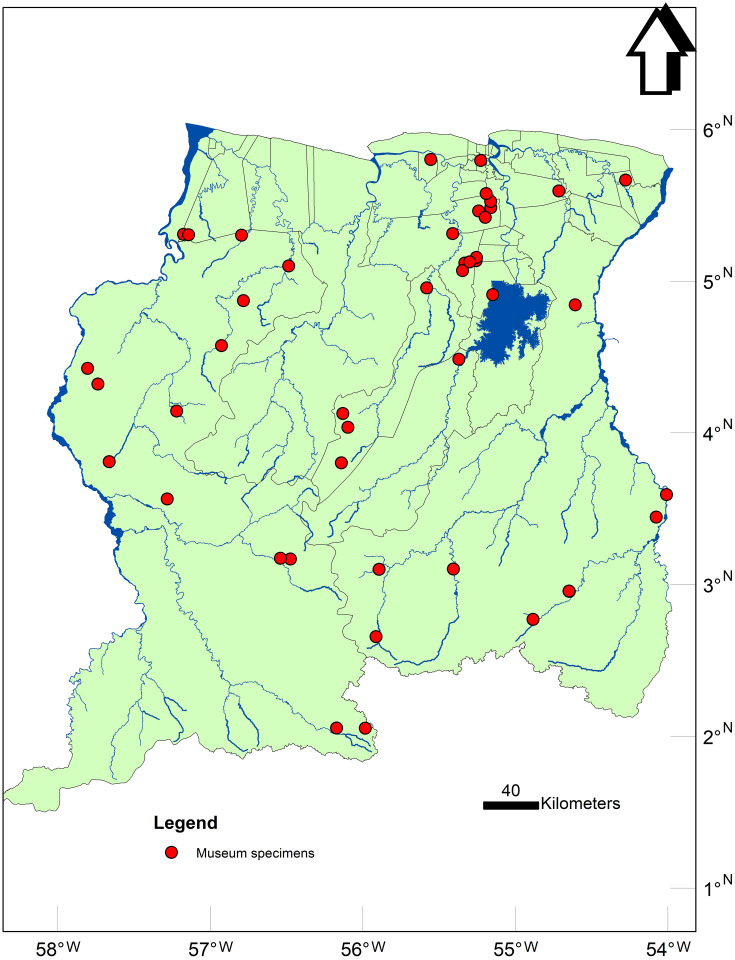
Bd sampling locations. Overview of all the locations (red dots) where *Batrachochytrium dendrobatidis* museum specimens were sampled in Suriname.

The NZCS houses approximately 2500 specimens of several amphibian species and is the only zoological institute in Suriname equipped to store specimens and disseminate knowledge on the diverse amphibian taxa present in Suriname. Species that were selected from the database for Bd testing were in the following families; Bufonidae, Dendrobatidae, Aromobatidae, Leptodactylidae, Ranidae and Pipidae. These families were selected due to past studies showing their members can be vulnerable to Bd infection (e.g., Bufonidae [15, 16] Dendrobatidae [17, 18] and Aromobatidae [9]) or have associations with water resources for parts of their life cycle (Leptodactylidae [19, 20] Ranidae [21] and Pipidae [8]), hence could encounter flagellated zoospores of the aquatic fungal pathogen Bd if it were present [22]. Temporal and spatial considerations also affected selection for sampling (Fig 1 and Table 1). Of the species that were selected in the families mentioned above some of the oldest specimens were collected in 1905 and consisted of 2 *Leptodactylus* specimens. Some specimens were chosen from just a few years back (2014 to 2016) targeting the location where collected. Waypoints on the map were selected based on their singularity of depicting a new geographic area. Although it might not be evident on the map for some areas, a selection for depicted waypoints was done using a minimum distance of 3 km from the nearest waypoint. The main purpose for this was to present all the different geographic areas sampled instead of cluttering the map with waypoints of every specimen. All species mentioned in the table are sorted starting with the oldest specimens collected on top.

**Table 1:** Table 1 gives an overview of the museum specimens from Suriname that were sampled for *Batrachochytrium dendrobatidis*. Specimens in the table are sorted on date collected in the field in ascending order.

NZCS #	Family	Genus	Species	Location	Date Collected	No of specimens
A572	Leptodactylidae	Leptodactylus	fuscus	Paramaribo neighbourhoods	May 17, 1905	1
A631	Leptodactylidae	Leptodactylus	rhodomystax	Paramaribo and neighbourhoods	May 17, 1905	1
A571	Leptodactylidae	Leptodactylus	fuscus	Charlesburg, Paramaribo	April 8, 1957	1
A435 & A438	Dendrobatidae	Dendrobates	tinctorius	Kappel Savanna	March 22, 1958	2
A425, A427	Dendrobatidae	Dendrobates	tinctorius	Basecamp Wilhelmina mountain	July 23, 1959	2
A433 & A434	Dendrobatidae	Dendrobates	tinctorius	Coeroeni island	August 12, 1959	2
A568	Leptodactylidae	Leptodactylus	bolivianus	Coeroeni island	August 12, 1959	1
A613	Leptodactylidae	Leptodactylus	mystaceus	Coeroenie island, expedition to Coeroeni	September, 1959	1
A632 & A633, A 634	Leptodactylidae	Leptodactylus	rhodomystax	Coeroeni island, expedition to Coeroenie	September, 1959	3
A220	Leptodactylidae	Leptodactylus	bolivianus	Kabelstation (flooded by creation of Brokopondo Lake)	May 17, 1961	1
A607 & A608	Leptodactylidae	Leptodactylus	longirostris	Kabelstation	May 17, 1961	2
A423 & A424	Dendrobatidae	Dendrobates	tinctorius	On path heading towards Juliana top	August, 1963	2
A428 & A429	Dendrobatidae	Dendrobates	tinctorius	Anapaike kondre, Lawa	November 18, 1963	1
A431 & A432	Dendrobatidae	Dendrobates	tinctorius	Bakhuis mountain	January, 1963	2
A219	Leptodactylidae	Leptodactylus	bolivianus	Mammadam soela	December 11, 1963	1
A620	Leptodactylidae	Leptodactylus	bolivianus	Camp Lucie river	August, 1963	1
A602	Leptodactylidae	Leptodactylus	knudseni	Camp Lucie rivier, expedition to Wilhelmina mountain	September, 1963	1
A614	Leptodactylidae	Leptodactylus	mystaceus	Camp Lucie river, expedition to Wilhelmina mountain	September, 1963	1
A615	Leptodactylidae	Leptodactylus	mystaceus	Pokigron	December 12, 1963	1
A621	Leptodactylidae	Leptodactylus	pentadactylus	In line with Lucie river	August 12, 1963	1
A622	Leptodactylidae	Leptodactylus	pentadactylus	Adampada expedition	September, 1964	1
A426	Dendrobatidae	Dendrobates	tinctorius	Raleighvallen, Central Suriname Nature Reserve	September, 1971	1
A570	Leptodactylidae	Leptodactylus	bolivianus	Baruba kreek, km 113, Kabalebo area district Nickerie	September 29, 1980	1
A573 to A575	Leptodactylidae	Leptodactylus	fuscus	Kopieweg, the first turning on the road, district Suriname	February 9, 1980	3
A179	Pipidae	Pipa	pipa	Avanavero falls	May 24, 1980	1
A347	Bufonidae	Atelopus	hoogmoedi	Road to Amotopo, km 80.5, district Nickerie	May 13, 1981	1
A348	Bufonidae	Atelopus	hoogmoedi	Road to Amotopo, km 39, 2000 m in line in North Western direction, district Nickerie	June 1, 1981	1
A566 & A567	Leptodactylidae	Leptodactylus	bolivianus	Road to Amotopo, km 25, Kabalebo area, district Nickerie	May 29, 1981	2
A600	Leptodactylidae	Leptodactylus	knudseni	Celos camping ground, Kabo, district Saramacca	February 4, 1981	1
A601, A605	Leptodactylidae	Leptodactylus	knudseni	Road to Celos proefcentrum Kabo, district Saramacca	February 7, 1981	2
A604	Leptodactylidae	Leptodactylus	knudseni	Road to Wittagron, 25 km from Celos proefcentrum Kabo, district Saramacca	February 6, 1981	1
A606	Leptodactylidae	Leptodactylus	knudseni	Road to Amotopo km 108.5, district Nickerie	May 8, 1981	1
A609 & A610	Leptodactylidae	Leptodactylus	longirostris	Celos camping Tonka, Kabo area, district Saramacca	February 4, 1981	2
A616 & A617	Leptodactylidae	Leptodactylus	mystaceus	Drempelkanaallijn, km 215–217.5, road to Amotopo	May 25, 1981	2
A569	Leptodactylidae	Leptodactylus	bolivianus	Camping ground G.M.D, Alaskakondre, Marowijne	August 28, 1982	1
A440	Aromobatidae	Allobates	femoralis	Alasakondre, camp G.M.D, Lawa, Marowijne	September 13, 1982	1
A603	Leptodactylidae	Leptodactylus	knudseni	Alaskakondre, district Marowijne	July 17, 1982	1
A635 & A636	Leptodactylidae	Leptodactylus	rhodomystax	Alaskakondre, upper Lawa, district Marowijne	July 17, 1982	2
A32	Aromobatidae	Allobates	femoralis	91 km upstream of Maratakka River, approx. 3 km from camp	November 12, 1987	1
A33	Leptodactylidae	Leptodactylus	bolivianus	40 km upstream of Maratakka River	November 15, 1987	1
A34	Leptodactylidae	Leptodactylus	bolivianus	91 km upstream of Maratakka River	November 6, 1987	1
A35	Leptodactylidae	Leptodactylus	bolivianus	125 km upstream of Maratakka River	November 8, 1987	1
A178	Leptodactylidae	Leptodactylus	fuscus	University of Suriname	October 9, 1987	1
A578 to A580	Leptodactylidae	Leptodactylus	fuscus	Dampoentong (near Groningen), district Saramacca	June 27, 1987	3
A59	Pipidae	Pipa	pipa	Dampoentong near Groningen, in recently deforested plots / agricultural land	June 27, 1987	1
A43 & A44	Dendrobatidae	Ameerega	trivittata	Dirt road to boat landing at Coesewijne River	April 13, 1988	2
A67	Dendrobatidae	Ameerega	trivittata	Sipaliwini River, Mouth of Akalapi creek	August 20, 1988	1
A75 t/m A77	Dendrobatidae	Dendrobates	tinctorius	Vier Gebroeders creek	August 28, 1988	3
A10	Aromobatidae	Allobates	femoralis	Zuid River, 15 km upstream from Kayser Mts. Airstrip	February 9, 1988	1
A11	Aromobatidae	Allobates	femoralis	Zuid River, 9 km downstream of Kayser Mountains Airstrip, on line transect 0.5 km North of river	February 13, 1988	1
A98	Ranidae	Rana	palmipes	Blanche Marie Falls	October 13, 1988	1
A1	Leptodactylidae	Leptodactylus	bolivianus	Zuid River, 9 km upstream of Kayser Mountains Airstrip	February 11, 1988	1
A2 & A3	Leptodactylidae	Leptodactylus	bolivianus	Kayser Mountains Airstrip	February 5, 1988	2
A69	Leptodactylidae	Leptodactylus	bolivianus	Vier Gebroeders creek, basecamp	August 22, 1988	1
A89 to A92	Leptodactylidae	Leptodactylus	bolivianus	Vier Gebroeders Mountains	August 28, 1988	4
A73	Leptodactylidae	Leptodactylus	fuscus	Sipaliwini savanna, Sipaliwini creek	August 25, 1988	1
A289, A297 & A298	Leptodactylidae	Leptodactylus	fuscus	Zanderij 1	June 25, 1988	3
A5, A20, A23, A24	Leptodactylidae	Leptodactylus	longirostris	Kayser Mountains Airstrip	February 5, 1988	4
A19, A22, A25	Leptodactylidae	Leptodactylus	longirostris	Savanna 3.5 km South- East of Kayser Mountains Airstrip	February 17, 1988	3
A68	Leptodactylidae	Leptodactylus	mystaceus	Sipaliwini River, Mouth of Apiego creek	August 18, 1988	1
A112	Pipidae	Pipa	pipa	Coesewijne River, km 19	November 1, 1988	1
A283	Leptodactylidae	Lithodytes	lineatus	South- East side of the Apalagadi, 350 m	August 16, 1989	1
A235, A262	Dendrobatidae	Dendrobates	tinctorius	South side of Apalagadi, Koewini creek, 300 m	August 14, 1989	2
A241	Dendrobatidae	Dendrobates	tinctorius	Palaime creek, (Sipaliwini savanna)	August 10, 1989	1
A237	Dendrobatidae	Dendrobates	tinctorius	2 km North-East of Sipaliwini airstrip	August 19, 1989	1
A263, A282	Aromobatidae	Ameerega	trivittata	South- East side of the Apalagadi, 480 m	August 16, 1989	2
A284, A285	Aromobatidae	Allobates	femoralis	Koewini creek, 300 m at the South side of the Apalagadi	August 17, 1989	2
A266	Dendrobatidae	Dendrobates	tinctorius	South- East side of the Apalagadi, 480 m	August 15, 1989	1
A147 to A149	Aromobatidae	Allobates	femoralis	Nickerie River, 159 km upstream of Wageningen, in line (transect)	March 11, 1989	3
A261, A264	Dendrobatidae	Dendrobates	tinctorius	South side of the Apalagadi, 350 m	August 14, 1989	2
A254	Leptodactylidae	Leptodactylus	mystaceus	Upper Coeroeni km 22, South Suriname	August 2, 1989	1
A258	Leptodactylidae	Leptodactylus	mystaceus	Koewini creek, 300 m at the South side of the Apalagadi	August 12, 1989	1
A340	Leptodactylidae	Leptodactylus	bolivianus	Kaboeri creek, West Suriname	September, 1990	1
A308	Leptodactylidae	Leptodactylus	fuscus	Para, Hannover	October 28, 1990	1
A305	Leptodactylidae	Lithodytes	lineatus	District Para, Loefbeek	July 23, 1990	1
A317	Pipidae	Pipa	pipa	Coropina creek, district Para, between Para river en Prinsie	October 24, 1990	1
A333 t/m A335	Aromobatidae	Allobates	femoralis	Kaboeri creek, West Suriname	September, 1990	3
A599	Pipidae	Pipa	Pipa	Prinsie, Coropina creek, district Para	October 8, 1990	1
A874	Leptodactylidae	Leptodactylus	pentadactylus	75 km upstream of Tepoe, between camp 3 and camp 4	September 1, 1991	1
A920 & A921	Bufonidae	Atelopus	hoogmoedi	Tepoe, camp 5	September 4, 1991	2
A779 to A781	Dendrobatidae	Dendrobates	tinctorius	Tepoe, Awalape creek, km 68	September 11, 1991	3
A906	Dendrobatidae	Dendrobates	tinctorius	Tepoe, camp 6, in line	September 8, 1991	1
A894a,b; A901a,b	Dendrobatidae	Dendrobates	tinctorius	Tepoe, camp 5	September 3, 1991	4
A931a,b,c,d	Dendrobatidae	Dendrobates	tinctorius	Tepoe, Awalapa creek km. 68	September 11, 1991	4
A875	Dendrobatidae	Dendrobates	tinctorius	101.5 km. upstream of Tepoe, by the mouth of the Peluli creek	September 4, 1991	1
A902, A911	Dendrobatidae	Dendrobates	tinctorius	Tepoe, line at the North side of camp 5	September 5, 1991	2
A744, A749 & A750	Bufonidae	Atelopus	hoogmoedi	Camp 5, 101.5 km upstream of Tepoe, at mouth of Peluli creek	September 5, 1991	3
A928	Leptodactylidae	Leptodactylus	pentadactylus	Tepoe, camp 5, 101.5 km. upstream	September 2, 1991	1
A929	Leptodactylidae	Leptodactylus	pentadactylus	Tepoe, camp 7, Ogoime	September 11, 1991	1
A664	Pipidae	Pipa	pipa	Zanderij 1, Savanna	July 22, 1992	1
A679	Aromobatidae	Allobates	femoralis	Palumeu airstrip, in the village	March 2, 1993	1
A764	Leptodactylidae	Leptodactylus	bolivianus	Akinto soela, Mapane area	June 27, 1993	1
A730, A731, A732	Leptodactylidae	Leptodactylus	bolivianus	Royal Hill	September 19, 1994	3
A693	Leptodactylidae	Leptodactylus	fuscus	Royal Hill, Gros Rosebel area in deep hole (5 m)	June 26, 1994	1
A694	Leptodactylidae	Leptodactylus	fuscus	Gross Savanna, North of Roma	June 24, 1994	1
A698	Leptodactylidae	Leptodactylus	knudseni	Koolhoven	June 20, 1994	1
A737	Leptodactylidae	Leptodactylus	knudseni	Maikaboeka creek	September 20, 1994	1
A717 to A721	Leptodactylidae	Lithodytes	lineatus	Royal Hill	August 10, 1994	3
A695	Leptodactylidae	Leptodactylus	longirostris	On the confluence of the Mindrineti river and the Maikaboeka creek	June 21, 1994	1
A696	Leptodactylidae	Leptodactylus	longirostris	Royal Hill, in mine shaft	June 17, 1994	1
A697	Leptodactylidae	Leptodactylus	longirostris	Mayo	June 24, 1994	1
A733	Leptodactylidae	Leptodactylus	longirostris	Royal Hill	August 10, 1994	1
A738	Leptodactylidae	Leptodactylus	pentadactylus	Royal Hill	August 10, 1994	1
A716	Aromobatidae	Allobates	femoralis	Koolhoven	September 26, 1994	1
A736	Leptodactylidae	Leptodactylus	rhodomystax	On the confluence of the Mindrineti river and the Maikaboeka creek	June 21, 1994	1
A736	Leptodactylidae	Leptodactylus	rhodomystax	Royal Hill	August 10, 1994	1
A759	Leptodactylidae	Leptodactylus	knudseni	Wane Hill 2, on the road	September 25, 1995	1
A888	Aromobatidae	Allobates	femoralis	Wane hill 2 (camp)	March 31, 1995	1
A739	Leptodactylidae	Leptodactylus	longirostris	Wane Hill 2 (slope)	March 29, 1995	1
A760	Leptodactylidae	Leptodactylus	longirostris	Wane Hill, camp	September 24, 1995	1
A767 to A769	Leptodactylidae	Leptodactylus	longirostris	Wane Hill 2, line AB	July 14, 1995	3
A879	Leptodactylidae	Leptodactylus	rhodomystax	Wane Hill 2	March 29, 1995	1
A908	Leptodactylidae	Leptodactylus	knudseni	Berlijn	May, 1997	1
A786	Leptodactylidae	Leptodactylus	bolivianus	Sipaliwini Airstrip	April 11, 1997	1
A794, A341	Leptodactylidae	Leptodactylus	fuscus	Sipaliwini Airstrip	April 15, 1997	2
A803, A805 & A806	Aromobatidae	Allobates	femoralis	Sipaliwini airstrip	April 14, 1997	3
A784	Leptodactylidae	Leptodactylus	rhodomystax	Sipaliwini Airstrip	April 16, 1997	1
A908	Leptodactylidae	Leptodactylus	knudseni	Berlijn	May, 1997	1
A844	Aromobatidae	Allobates	femoralis	Ulemari, 99 km upstream of confluence with the Litani	April 12, 1998	1
A843	Leptodactylidae	Leptodactylus	knudseni	Ulemari, 13 km upstream of confluence with the Litani	April 5, 1998	1
A860	Dendrobatidae	Dendrobates	tinctorius	Ulemari	April 22, 1998	1
A851	Aromobatidae	Allobates	femoralis	Ulemari, 39 km upstream of confluence with the Litani	April 30 & May 1, 1998	1
A863 & A864	Dendrobatidae	Dendrobates	tinctorius	Oranje mountain, left tributary, Ulemari	April 15, 1998	2
A873	Leptodactylidae	Leptodactylus	rhodomystax	Ulemari	April 22, 1998	1
A932a,b,c	Dendrobatidae	Dendrobates	tinctorius	Sipaliwini, Tepoe	August, 1999	3
A904	Pipidae	Pipa	pipa	Maykabuka tributary	February 10, 1999	1
A985	Leptodactylidae	Leptodactylus	bolivianus	Brownsberg, on the mazaroni road	January 28, 2009	1
A1016	Dendrobatidae	Ameerega	trivittata	Sipaliwini Apalagadi	April 19, 2014	1
A1034	Dendrobatidae	Ameerega	hahneli	Sipaliwini	June 27, 2014	1
A1063	Dendrobatidae	Ameerega	hahneli	Sipaliwini Apalagadi	April 18, 2014	1
A1064	Dendrobatidae	Ameerega	trivittata	Sipaliwini community	April 15, 2014	2
A1076 & A1077	Dendrobatidae	Ameerega	trivittata	Nassau trail 7	December 15, 2014	2
A1000a, A1000b	Leptodactylidae	Leptodactylus	longirostris	Sipaliwini	April 22, 2014	2
A1122	Dendrobatidae	Ameerega	trivittata	Bakhuis Mountains, near camp	April 26, 2015	1
A1139 & A1140	Dendrobatidae	Dendrobates	tinctorius	Bakhuis Mountains, Low creek	April 28, 2015	2
A1175 & A1176	Pipidae	Pipa	pipa	Voltzberg, raleighvallen	January 24, 2016	2
A430	Dendrobatidae	Dendrobates	tinctorius	Basecamp on the foot of the Emma mountains, appr. 350 m high	date unknown.	1

### Specimen sampling

Amphibian specimens used for this study were selected by querying the NZCS database.

To swab for Bd we followed the same procedure as Rodriguez et al. [23] where each individual frog was first rinsed with clean uncontaminated 70% ethanol and allowed to dry on a clean sheet of tissue paper. We then swabbed each specimen using Medical Wire swabs as per the standard swabbing methods described by in Hyatt et al. [24] and Cheng et al. [13]. We swabbed and pooled up to eight specimens to minimize the cost of lab analyses keeping swabs of the same species in one vial as much as possible. The vial number on each vial was linked to the NZCS no of the specimens that were swabbed with that swab. Pooling swabs or swabbing multiple specimens has become an accepted method of verifying Bd in presence absence studies [24, 25]. After swabbing the specimens, swabs were cut to desired length and placed in sterile 1.5ml screw top centrifuge tubes with O rings. Swabs were allowed to dry completely before the tubes were closed and stored in a refrigerator with a temperature of 4°C until shipped to be processed.

### Sample preparation

All swabs were processed using the following procedure. One ml of 70% ethanol diluted to 70% final concentration with deionized H_2_O) was added in the lab to each sample tube. After vigorous mixing the liquid to dislodge any zoospores/skin tissue from the swabs, the entire volume from each sample was transferred in two different microfuge tubes. The tubes were spun in a microcentrifuge at ~16,000 x G for 3 minutes. Next, the supernatant was drawn off and discarded since B.d zoospores are negatively buoyant in 70% ethanol and therefore will pellet upon centrifugation. Lysis buffer (180 uL of Qiagen ATL buffer + 20 uL Qiagen Proteinase K) was added to the tubes and any pellet present was resuspended by vortexing. Ten μg of carrier DNA was added to the lysis buffer. Total DNA was extracted from all samples using a silica membrane spin-column DNA purification procedure (Qiagene DNeasy, blood and tissue kit).

#### qPCR assay

The sample DNAs were assayed for the presence of the *Batrachochytrium dendrobatidis* ribosomal RNA Intervening Transcribed Spacer (ITS1) region by 45 cycle PCR amplification using a qPCR assay developed at Pisces and a Stratagene MX4000 real-time PCR instrument. Primer and probe base sequence are as follow:

Primer Forward: 5' TGGATGGGAGTTTTATTGATGTGTA

Reverse: 5' TCGTGACATATGGCACACTGTATT and the probe 5'-FAM -TGG AAT GAC CCA TTG TT-BHQ1 plus. The reaction master mix contains ROX as passive reference dye for normalizing variations in individual reaction total volumes, and a VIC-labeled internal positive control (IPC) (Life Technologies TaqMan Positive Control, catalog #4308323) to detect PCR inhibition. The detection sensitivity of this assay is three target sequence molecules (approximately 0.02 zoospore equivalents). Each PCR run included the following controls: Positive DNA: DNA prepared from a plasmid constructed at Pisces containing the *B*. *dendrobatidis* ribosomal RNA Intervening Transcribed Spacer (ITS1) region. Serial ten-fold dilutions of this plasmid DNA from 2.9 x 10^6^ to 2.9 x 10^0^ molecules per reaction were used to generate the standard curve. Serial dilutions controls were done since these spanned the Ct values observed for all positive samples observed while using this assay.

#### Control

Water in place of template DNA. This reaction remains uncapped during addition of sample DNA to the test reactions, and serves as a control to detect contaminating DNA in the PCR reagents or carryover of positive DNA during reaction set-up.

## Results

Using the 0.02 threshold for any sample that gave clearly observable, exponential (log-linear) fluorescent signal increase we found no museum specimens to be positive for the chytrid fungus. However, any weak or questionably positive samples were retested in a second independent qPCR run. Only samples which retested positive in this second run were scored as positive. In the study herein all the samples were tested negative. The specimens used for swabbing also showed no aberrant skin condition to indicate a possible chytrid infection.

## Discussion

The negative results for the museum specimens tested in this study supports that Bd has not infected any frogs in Suriname. Many South American countries have had specimens tested positive for this fungus including countries bordering Suriname. The data presented herein and the specimens sampled in the field by the author gives a first country wide overview of this chytrid fungus sampling and as such serves as a baseline study for future Bd presence/absence research. The inclusion of museum specimens in this chytrid sampling overview for Suriname, apart from the historical point of view enabled us also to sample specimens that were not sampled in the field survey due to a lack of funds to be able to visit those places. The research presented herein of the 15 different species sampled added to the number of species sampled during the field survey gives us a total of 52 frogs species sampled for Suriname and a total of 555 specimens. One particular reason for the Bd free status of Suriname could probably be due to the relatively high mean temperature of Suriname which varies between 26.2°C for the coldest month to 28.2°C for the warmest time period [26] thus making conditions not favorable for Bd to get established [27, 28]. However, the inhibition by temperature should not be ruled out completely as lowland sites have also been reservoirs of species carrying the pathogen [29, 30] so chances are that in due time and with few existing precautions chytridiomycosis caused by Bd might eventually set foot in Suriname. The wide eastern and western river border of Suriname could be yet another reason that this chytrid fungus has not yet reached here. Evidence that Bd infection spreading into different countries seems to be mostly coupled with human activities [31] and that possibly infected animals or substrate with the zoospores could make it into Suriname. Although this fungus has spread due to the pet trade in many countries [32] it is highly unlikely that the amphibian pet trade in Suriname will develop to such an extent that frogs will be imported from other countries to keep as pets. In the same vein it is not envisaged that frogs will be imported from other countries to be consumed or bred here to serve as part of the Surinamese diet. Ornamental fish however are imported in Suriname and have shown to be carriers of this chytrid fungus [33].

Regular surveys sampling for Bd in Suriname should be a mandatory activity to constantly monitor the status of this pathogen.
